# Phase variation of a Type IIG restriction-modification enzyme alters site-specific methylation patterns and gene expression in *Campylobacter jejuni* strain NCTC11168

**DOI:** 10.1093/nar/gkw019

**Published:** 2016-01-18

**Authors:** Awais Anjum, Kelly J. Brathwaite, Jack Aidley, Phillippa L. Connerton, Nicola J. Cummings, Julian Parkhill, Ian Connerton, Christopher D. Bayliss

**Affiliations:** 1 Department of Genetics, University of Leicester, Leicester LE1 7RH, UK; 2 Division of Food Sciences, School of Biosciences, University of Nottingham, Sutton Bonington LE12 5RD, UK; 3 The Sanger Institute, Wellcome Genome Campus, Hinxton, Cambridge CB10 1SA, UK

## Abstract

Phase-variable restriction-modification systems are a feature of a diverse range of bacterial species. Stochastic, reversible switches in expression of the methyltransferase produces variation in methylation of specific sequences. Phase-variable methylation by both Type I and Type III methyltransferases is associated with altered gene expression and phenotypic variation. One phase-variable gene of *Campylobacter jejuni* encodes a homologue of an unusual Type IIG restriction-modification system in which the endonuclease and methyltransferase are encoded by a single gene. Using both inhibition of restriction and PacBio-derived methylome analyses of mutants and phase-variants, the *cj0031c* allele in *C. jejuni* strain NCTC11168 was demonstrated to specifically methylate adenine in 5΄CCCGA and 5΄CCTGA sequences. Alterations in the levels of specific transcripts were detected using RNA-Seq in phase-variants and mutants of *cj0031c* but these changes did not correlate with observed differences in phenotypic behaviour. Alterations in restriction of phage growth were also associated with phase variation (PV) of *cj0031c* and correlated with presence of sites in the genomes of these phages. We conclude that PV of a Type IIG restriction-modification system causes changes in site-specific methylation patterns and gene expression patterns that may indirectly change adaptive traits.

## INTRODUCTION

*Campylobacter jejuni* is a major cause of food-borne gastroenteritis in humans with more than 400 million cases of Campylobacteriosis reported annually worldwide (1). Currently, the disease burden exerted by *Campylobacters* in the UK is higher than for any other foodborne pathogen (2) with chickens being the main source of infection in over 80% of disease cases (3). *Campylobacters* are present in a very high fraction of farmed chickens with significant numbers of bacteria in each colonized bird (4,5). Rapid micro-evolution may be a major mechanism that facilitates persistent colonization of and rapid transmission between individual birds. One of the characteristic mechanisms for enabling rapid adaptation is phase variation (PV) mediated by hypermutable simple sequence repeats (6). Most of phase-variable genes of *C. jejuni* encode surface proteins or enzymes that modify host surface structures (7). However one of these phase-variable genes encodes a restriction modification (RM) system that has the potential to act as a stochastically-regulated modifier of expression of other genes or mediate alternation between phage susceptible and resistant states (8).

PV is an adaptive process characterized by high frequency, reversible ON/OFF switching of gene expression that is stochastic in nature and gives rise to heterogeneous population (9). The main mechanism of PV in *C. jejuni* involves insertion or deletion of repeat units during genome replication in polyG or polyC repeat tracts located in the reading frames of genes. In a recent survey, each *C. jejuni* genome was found to contain between 12 and 29 polyG/polyC tracts capable of mediating PV (8). Most of these phase-variable loci encode enzymes that modify the glycan components of the flagella, capsule and lipooligosaccharide. One of the tracts was present in a putative Type IIG restriction-modification system. This is a general paradigm with phase-variable Type I and III RM systems being found in other host adapted bacterial pathogens including *Helicobacter pylori, Neisseria meningitidis, Neisseria gonorrhoeae* and *Haemophilus influenzae* (6).

The three major classes of RM systems (i.e. I, II and III) are distinguished by the composition and nature of the recognition sequences, position of cleavage, requirements for co-factors and subunit composition (10). A Type IIG RM system is a subclass of the Type II RM systems in the majority of which the endonuclease (ENase) and methyltransferase (MTase) activities are fused into a single polypeptide chain rather than being present as separate enzymes. These Type IIG enzymes methylate only one strand within either a symmetric or asymmetric recognition site while the endonuclease activity occurs 14–21 bp away from the recognition site in a 3΄ direction (11). This biochemical property of Type IIG RM systems is similar to that of Type IIS and Type III RM systems. Two of the most highly characterized prototypical Type IIG enzymes are BpuSI and Eco57I (12,13). However, REBASE (rebase.neb.com/rebase/rebase.html; 14) predicts the existence of 6400+ putative Type IIG RM enzymes with 100+ recognition sites and a presence in a vast range of bacterial species.

RM systems are regarded as defensive weapons of pathogenic bacteria preventing infection by phages or invasion by foreign DNA (15). A putative role in epigenetic gene regulation through methylation-dependent control of the interactions of DNA binding regulatory proteins with their cognate recognition sites was suggested by analogy with the Dam methylase, a known epigenetic regulator of bacterial virulence gene expression (16–18). The role of RM-mediated epigenetic regulation was demonstrated by showing that ON/OFF PV of the *H. influenzae modA* gene was correlated with altered expression of several genes (19). This gene encodes the methyltransferase component of a Type III RM system whose PV-dependent co-regulated genes were termed the phasevarion. Subsequently, Type III RM systems in three other human pathogens, *H*.*pylori, N. meningitidis* and *N. gonorrhoeae* (20,21) and a Type I system in *Streptococcus pneumoniae* were found to have phasevarion activities (22). The alterations in expression of a ‘regulon’ are predicted to be mediated through differential methylation of promoter elements or other regulatory sequences. Critically, phasevarions include genes mediating resistance to stress and virulence phenotypes leading to the suggestion that phase-variable RM systems may be a common mechanism for regulation of virulence and stress survival in a wide range of bacterial pathogens.

Most *C. jejuni* strains contain one Type I RM and four Type II systems, one of which is a Type IIG enzyme (23,24). This Type IIG RM system exhibits significant sequence variation between strains in the domain presumed to encode the target recognition domain (TRD). In agreement with this hypothesis, strains vary in the methylation site associated with this gene. In strain NTCT11168, the homologue of this Type IIG gene, *cj0031*, contains a polyG tract and undergoes PV both *in vivo* and *in vitro* (8). This observation of major shifts in the bacterial population from the OFF to ON expression state of *cj0031*, during passage of *C. jejuni* in chickens, combined with similarities in the mechanism of methylation to the phasevarion-associated Type III RM systems, suggested that PV of *cj0031* may confer fitness advantages on the bacterial population, due to alterations in expression of multiple genes mediated by changes in global methylation. To test this hypothesis, we investigated the functions, methylome, global expression patterns and phenotypic variation of mutants and phase-variants of *cj0031* in *C. jejuni strain* NCTC11168.

## MATERIALS AND METHODS

### Bacterial strains and growth conditions

Two variants of *C. jejuni* strain NCTC11168 were used in the present study: a motile but non-swarming variant (11168); and a chicken-adapted hypermotile variant (CH11168). The *C. jejuni* strains were grown on Mueller-Hinton agar (MHA, Oxoid, UK) supplemented with vancomycin (10 μg/ml Sigma Aldrich, UK) and trimethoprim (5 μg/ml Sigma Aldrich) in a VA500 Variable Atmosphere Incubator (Don Whitley, UK) providing 4% oxygen (v/v), 10% carbon dioxide (v/v), 86% nitrogen (v/v) and a temperature of 42°C. *Esherichia coli* strain DH5α was used for cloning and was cultured at 37°C on Luria agar plates or in Luria broth containing appropriate antibiotic as required; ampicillin, 50 μg/ml; kanamycin, 50 μg/ml; and chloramphenicol, 10 μg/ml.

### Phylogenetic analysis

The *cj0031* gene sequence was downloaded from the NCBI database, and then used in a BLAST search to identify homologues of this gene and of the allelic variant, *A911_00150*, present in *C. jejuni* PT14 (25). These sequences were submitted to the Phylogeny.fr platform in order to construct a phylogenetic tree. This platform uses MUSCLE for the alignment of the submitted sequences, PhyML for tree building and TreeDyn for tree rendering (26). For building phylogenetic tree of *A911_00150*, the default parameters set for MUSCLE, PhyML and TreeDyn were applied.

### Isolation of ON and OFF phase-variants

Serial dilutions of a single colony of *C. jejuni* NCTC11168 strain were plated onto MHA plates and incubated for 3 days under micro-aerobic conditions at 42°C. A total of 110 colonies were randomly selected and sub-cultured. Boiled lysates were prepared in 100 μl of distilled water for DNA amplification. The repeat tract of *cj0031* was amplified using two primers, 0031F-Fam and 0031R and then analysed by GeneScan as described elsewhere (8). A total of 75 isolates had a tract length of G10 (an OFF-phenotype) and 13 had G9 (an ON phenotype). The tract lengths of three isolates of each type were confirmed by dideoxy sequencing.

### Identification of the Cj0031 methylation site by inhibition of restriction

Changes in the restriction patterns of chromosomal DNA extracted from phase variants and mutants was identified as follows. Chromosomal DNA was prepared using either a phenol extraction/caesium chloride method or a DNeasy extraction kit (Qiagen). Two micrograms of DNA was digested for 2 h at 37°C with 20–40 units of a range of methylation sensitive restriction enzymes. The resulting fragments were resolved on 1% agarose gels at 80V for 2–3 h, and visualized under UV illumination. Southern blot and hybridization (27) were performed using DIG-labelled (Roche) polymerase chain reaction (PCR) products as probes. These probes were generated using primers listed in Supplementary Table SI and were designed to contain one or more of the relevant restriction sites within chromosomal fragments of 0.5–10 kb. Products were visualized using a DIG luminescent detection kit (Sigma-Aldrich, UK) and autoradiography.

### Construction of deletion and complementation mutants of cj0031 in *C. jejuni* variants 11168 and CH11168

For the deletion mutant, the M0031-up fragment was amplified from upstream of *cj0031* using primers 0031–2108-F-pst1 and 0031–2832-R-2832-Bam (Supplementary Table SI). A second fragment (S0031-down) having a size of 621 bp was amplified from the downstream region of *cj0032* using primers 0032-Bam-F and 0032-R (Supplementary Table SI). These PCR products were cloned separately into pGEM-TEasy to create pGEM-M0031-up and pGEM-S0031-down and then these recombinant plasmids were digested with PstI and BamHI to release the M*0031*-up fragment or BamHI and EcoRI to release the S*0031*-down fragment, respectively. These fragments were subcloned into pUC18, digested with PstI and EcoRI, in a three-way ligation reaction to obtain pUC18-M0031-up-S0032-down. This plasmid was linearized with BamHI and ligated to a 1.4 kb fragment containing a kanamycin resistance cassette and recovered from pJKM30 by BamHI digestion. The resulting plasmid, pUC18-*cj0031*::*kan*, was recombined into competent cells from 11168 and CH11168, and transformants were verified by PCR amplification and sequencing.

The pCfdxA complementation vector (28) enables recombination into *cj0046*, a pseudogene of *C. jejuni* NCTC11168. The *cj0031* gene was amplified with primers N-cj0031-F and N-cj0031-R (Supplementary Table SI). The pCfdxA vector was cleaved with BsmBI (Esp3I), which generates Esp3I and NcoI overhangs and this was ligated to PCR products cut with Esp3I and NcoI. The resulting recombinant vector pCfdxA0031 was transformed into the *C. jejuni* mutant strains to generate the complementation constructs NCTC11168Δ*0031*::*kan*::*0031c* and CH11168Δ*0031*::*kan*::*0031c*. PCR and sequencing reactions were performed to verify the integrity of *cj0031* and the orientation relative to the CAT promoter in pCfdxA0031 using three pairs of primers: CAT-INV-F, 0031–2108-R; 0046-INVF, 0032-R; and CAT-INV-F, 0031–2832-R.

### Adhesion and invasion assays

*Campylobacter* strains CH11168-Δ*0031*::*kan*, CH11168-*cj0031*-ON (G9) and CH11168-*cj0031*-OFF (G10) were cultured for 3 days on MHA plates with antibiotics and then a sweep of cells was inoculated into MHB (without antibiotics). After overnight incubation under shaking conditions, the OD_600_ of the culture was adjusted to 0.5 (∼2 × 10^9^ cells) prior to adding 10 μl (1 × 10^7^ cells) into selected wells of a 24-well tissue culture plate containing a confluent monolayer of Caco-2 cells. After 4 h incubation at 37°C with 5% CO_2_, media was removed and wells were gently washed twice with 2 ml of phosphate buffered saline (PBS). After washing, 1 ml of PBS/1% Triton X100 was added to each well and incubated for 10 min at room temperature to allow for lysis of the Caco-2 cells. Lysates were mixed and then subject to serial dilution in PBS in triplicate. A 50 μl aliquot of each dilution was plated onto an MHA plate and, after 2–3 d, CFUs were counted and the number of adherent bacterial cells was calculated.

For the invasion assays, the Caco-2 monolayer were challenged with 2 × 10^7^ bacterial cells and incubated for 4 h at 37°C. After washing the monolayers, fresh media containing 200 μl gentamycin was added to each well and incubation continued for a further 4 h. Each well was then processed as described above in and the numbers of invasive bacterial cells were determined from duplicate serial dilutions.

### Measurement of biofilm formation

Biofilm formation (29) was performed using *C. jejuni* strains, grown overnight in MHB under shaking conditions, which were then diluted in MHB broth to give a final OD_600_ of 0.025 (∼ 2.5 × 10^7^ CFU). One ml of this suspension was added to each well of a 24-well polystyrene plates (Corning). Plates were incubated for either 48 or 72 h. Following incubation, the media was discarded and wells were washed twice with distilled water. Plates were dried at 55°C for 30 min and then 1 ml of 0.1% crystal violet (CV) was pipetted into each well followed by 10 min incubation at room temperature. The CV solution was removed and wells were washed twice with water followed by drying at 55°C for 15 min. Bound CV was solubilized in 1 ml of 80%- ethanol-20% acetone. The absorbance at 570 nm of a 100 μl aliquot was determined using a micro-plate reader (BMG-Labtech) as a measure of the biofilm mass.

### Motility assay

Bacterial cells harvested from MHA plates were suspended in MHB and the optical density at 600 nm was adjusted to 0.45 (∼1 × 10^9^ CFU/ml). A 2 μl aliquot of this bacterial suspension was stabbed into a 0.4% MHA plate and incubated for 48–72 h.

### Testing the efficacy of plaque formation of phages on *C. jejuni* host strains

Propagation of bacteriophages (Supplementary Table SII) was carried out using the following host *C. jejuni* strains: PT14, HPC5 and 11168 (30) as previously described with slight modification (31). Briefly, the *C. jejuni* host strains were sub-cultured on MHA plates for 24 h under micro-aerobic conditions, bacterial cells were scrapped off and suspended in 10 ml of 10 mM MgSO_4_ and adjusted to a cell density of ∼1 × 10^9^ CFU/ml. A 500 μl aliquot of cell suspension was added into molten NZCYM top agar (0.6% agar w/v), and poured on to the basal NZCYM plates (1.2% agar w/v). Serial 10-fold, dilutions of the phages were prepared in SM buffer and applied as 10 μl droplets, in triplicate, on to the surface of the NZCYM overlay plates. Once the droplets had been absorbed, the plates were incubated for 24 h, under micro-aerobic conditions, at 42°C. The plaques were counted and used to calculate the plaque forming units per ml (PFU/ml).

### SMRT sequencing and detection of the methylome

Genomic DNA was prepared using a DNeasy extraction kit (Qiagen) and sequenced on the Pacific Biosciences RSII machine according to standard manufacturer's conditions. Template preparation used kit version 3.0, polymerase binding used version P4 and sequencing reagents were version C2. Data was captured using 3 h movies. Each sample was sequenced on two single molecule real time sequencing (SMRT) cells, to give genome coverage of >200-fold per sample. Data has been deposited in the European Nucleotide Archive, and accession numbers are given in Supplementary Table SIII. The genomic positions of each methylation site motif for Cj0031 were determined by a word search.

The relative positions of each 5΄CCYGA sequence in the genome sequence of *C. jejuni* strain NCTC11168 were detected using a script to determine position relative to the nearest coding sequence.

### Analysis of gene expression by RNASeq

Total RNAs were extracted from three independent log phase cultures of CH11168 *cj0031*-ON and the deletion mutant CH11168-Δ*cj0031*::kan using the TRIzol^®^ Max™ Bacterial Isolation kit with Max™ Bacterial Enhancement Reagent (Invitrogen, Paisley, UK) according to the manufacturer's instructions, before ethanol-precipitation and purification using the RNeasy^®^ Mini kit of Qiagen (Crawley, UK) according to the manufacturer's instructions. On-column DNase treatment was included during the purification using the RNase-free DNase set (Qiagen). The purified total RNAs were assessed for quality and rRNA depletion using an Agilent 2100 Bioanalyzer (Agilent Technologies Inc., South Queensferry, UK) with Prokaryote Total RNA Nano series II software, Version 2.3. RNA samples with an RNA Integrity Number (RIN) of 7.0 and above were selected for cDNA library preparation. Total RNA yields were determined using an ND-1000 spectrophotometer (NanoDrop Technologies). Ribosomal RNAs were depleted using Ribo-ZeroTM rRNA removal kit for Gram-negative bacteria (Epicentre Biotechnologies, Madison, WI, USA), from which cDNA libraries were prepared with the TruSeq™ RNA sample preparation kit (Illumina, San Diego, California). Each library was indexed with a unique identification adapter sequence before 15 cycles of PCR enrichment. The libraries were validated using the MultiNA analyzer (Shimadzu Corporation) and were normalized to ∼10 nM. The indexed libraries were pooled and loaded onto a single lane of an Illumina HiSeq 2000 flowcell at a final concentration of 7 pM.

Raw sequence reads were imported into the CLC Genomics Workbench 6.0 package where filtering removed low quality reads, trimmed the TruSeq™ adapter index and removed duplicate reads. Sequence reads were mapped to the *C. jejuni* NCTC11168 genome sequence (accession number AL111168.1). Quality control of the sequences derived from replicate cDNA libraries were analysed by principal component analysis. Differential expression was determined using normalized RPKM (reads per kilobase of transcript per million; 32) in conjunction with Baggerly's test statistic (33). To control the error rate, *P*-values were calculated using the False Discovery Rate (FDR; 34). Genes with a fold change of 1.5 or more and a corrected *P*-value of <0.05 were considered to exhibit differential expression, and represented using Circos diagrams (35).

### Real time qRT-PCR

Total RNAs were converted to cDNA using the Omniscript cDNA synthesis kit (Qiagen) according to the manufacturer's protocol. Specific primers for qRT PCR were designed with lengths of 18–24 nt with melting temperatures at 57°C. Aliquots of cDNA were used as the template for qRT PCR. An optical 46 well microtiter plate (Applied Biosystems) was used with a 20 μl reaction volume consisting of Power SYBR^®^ Green PCR master mix (Life Technologies), 50 nM gene specific primers and the template. An ABI Prism 7000 sequence detector (Applied Biosystems) was programmed for an initial set up of 30 s at 95°C, followed by 40 cycles of 15 s at 95°C and 1 min at 57–58°C. SYBR^®^ Green detects double stranded DNA. A melt curve was obtained from a first step starting from 50–95°C to control specificities of quantitative PCR reaction for each primer pair. Cycle threshold (CT) values were determined using Step one software version 2.0 (Applied Biosystems). The comparative threshold cycle method was used to calculate change (n-fold) where samples were normalized to the internal control product of the *sod*B gene (36), which showed no change in expression levels between CH11168 *cj0031*-ON and CH11168-Δ*cj0031*::kan. Reactions were performed in triplicate. The fold changes were calculated using the 2ΔΔCt method. The primer pairs utilized in the qRT PCR experiments to determine the change in expression for selected genes are given in Supplementary Table SIV.

## RESULTS

### Distribution and conservation of Cj0031 homologues

**Figure 1.**
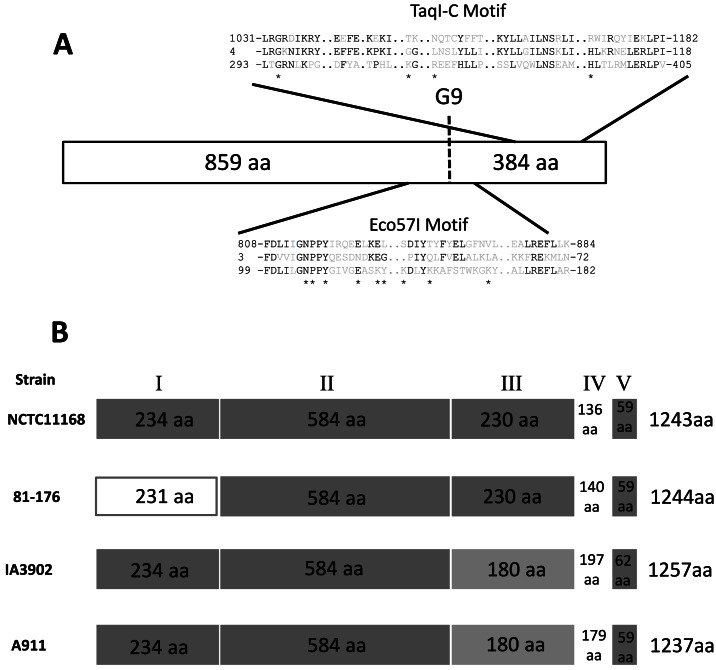
Alignments and sequence conservation of Cj0031 homologues. (**A**) The positions and sequences of the conserved domains of the amino acid sequence of Cj0031 (top sequence) are shown in comparison to M.TaqI (2IBS_A; GI:155126; bottom sequence) and the consensus sequences of the Eco57I (pfam07669; middle sequence) and TaqI_C domains (pfam12950; middle sequence). The amino acids marked with an asterisk are involved in DNA interactions, specific and non-specific, as shown by Goedecke *et al.* for M.TaqI (51). The position of the phase-variable G-tract in Cj0031 is marked as G9. (**B**) Representation of the conserved regions in Cj0031 and homologues from *Campylobacter jejuni* strains whose methylomes have been characterized. The dark grey boxes indicate >90% homology between sequences. The light grey regions indicate >90% homology between the two sequences but divergence from the other two sequences.

The *cj0031* gene of *C. jejuni* strain NCTC11168 is 3732 bp in length and contains a polyG tract of nine residues at position 2573 bp. Deletions or insertions in this tract can separate the gene into two reading frames (called *cj0031* and *cj0032* in the original annotation of the genome sequence). The *cj0031* amino acid sequence (1243 amino acids) has homology to known Type IIG RM enzymes with conserved domains present on either side of the polyG tract that includes conservation of amino acids involved in specific and non-specific DNA interactions in the M.TaqI crystal structure (Figure 1). An unusual feature of these systems is that the methyltransferase and endonuclease activities are fused into a single reading frame. PV mediated by alterations in the repeat tract are predicted to simultaneously abrogate both activities, i.e. methylation and restriction, reducing the toxic effects associated with inactivation of other Type II RM systems.

**Figure 2.**
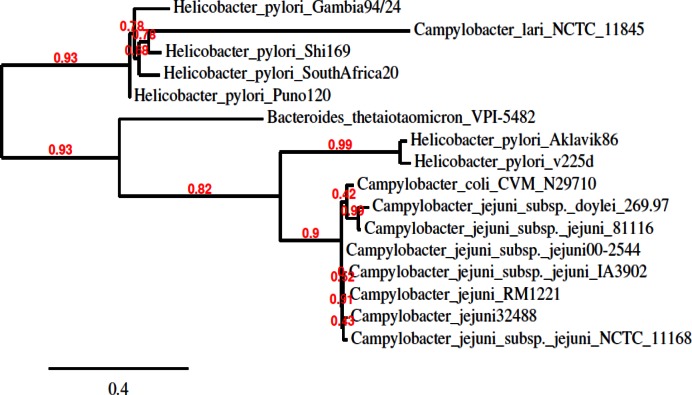
Phylogenetic tree showing relationship of *cj0031* with orthologues in other species. The tree was generated by using Phylogeny.fr software. The alignment was of the *cj0031* amino acid sequence with homologues identified through a BLAST analysis. The sequences were aligned using MUSCLE and entered into PhyML for tree building followed by TreeDyn for tree rendering.

Orthologues of Cj0031 were found in several other species including a distant association with a putative RM system of the cyanobacteria, *Microcystis aeruginosa* (data not shown). Homologues from *Campylobacter* and *H. pylori*, the closest neighbour of *C. jejuni*, fell into two groups, separated by homologues in more distant organisms, suggestive of the possibility of acquisition by horizontal transfer (Figure 2). Homologues were present in other *Campylobacters* including all other *C. jejuni* strains. A notable feature was the lack of a repeat tract in many of the other strains indicating that PV only occurs in a limited number of alleles and is not a universal feature of this gene. For example, strain 81–176 had an interrupted repeat tract (4G-A-4G) that will effectively prevent PV in this allele. Alignments were performed between the *cj0031* nucleotide and amino acid sequences and those of homologues from the four *C. jejuni* strains whose methylomes have been determined (37–39). Strain F38011 exhibited a ∼300 bp deletion in the central portion of the gene, expected to abrogate methylation activity and explaining the lack of methylation at sites recognized by this protein in the genome. The other three strains showed high levels of conservation in the N-terminal (regions I and II) and C-terminal regions (region V) with the exception of significant divergence in amino terminal region 1 of strain 81–176. The sequences diverged into two classes in region III and were all highly divergent in region IV suggesting the presence of a variable domain responsible for observed differences in site-specific methylation by each of these Cj0031 homologues.

### Characterization of the recognition site of Cj0031 by inhibition of restriction

**Figure 3.**
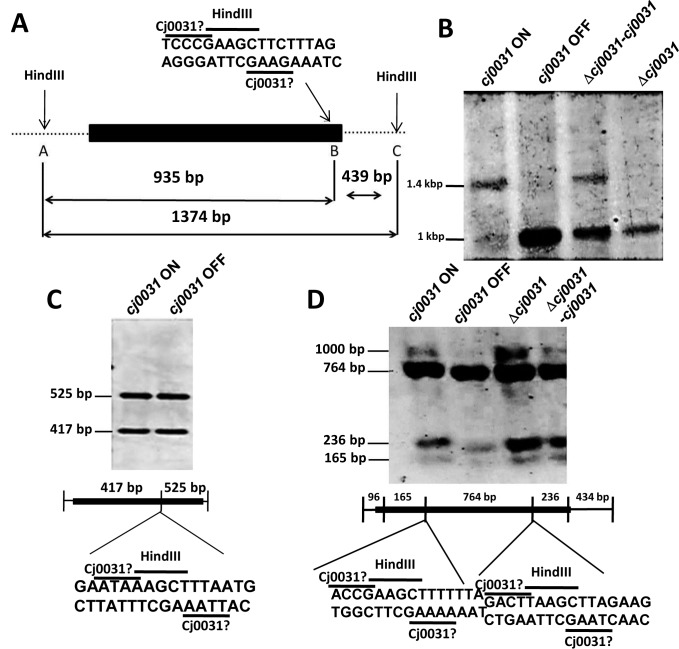
Identification of the methylation target site for Cj0031. Chromosomal DNA was extracted from ON and OFF phase variants (i.e. 11168-*cj0031*-ON and 11168-*cj0031*-OFF, respectively), a deletion mutant (11168-Δ*cj0031*::kan) and a complementation mutant (11168Δ*cj0031-cj0031*) of *cj0031* in *Campylobacter jejuni* strain NCTC11168. DNA was subjected to restriction with HindIII followed by probing of Southern blots with region specific probes. (**A**) Schematic diagram of the HindIII recognition sites (A, B and C) and overlapping putative Cj0031 recognition site present in Probe III. The figure indicates the sizes of fragments generated by complete and partial HindIII digestion with the latter resulting from protection of site B by Cj0031. (**B**) Analysis of HindIII restriction fragments using probe III. Inhibition of restriction is observed in the ON phase variant and complementation strain but not the OFF variant or deletion mutant. (**C**) Analysis of HindIII restriction fragments using probe I. (**D**) Analysis of HindIII restriction fragments using probe PSR-HindIII-CCG. This probe covers a region in which the 5΄CCGAA and 5΄CGAAG sequences (present in Probe III) overlap with the HindIII sequence but are not protected against HindIII digestion. Black rectangles, position of probes; Cj0031?, putative Cj0031 recognition site; HindIII, recognition site for HindIII.

A previous attempt to identify the methylation site of Cj0031 was compromised by use of a strain with an OFF number of repeats in *cj0031* (37). To overcome this difficulty, both ON and OFF phase-variants were isolated from a population of cells for *C. jejuni* strain NCTC11168 (see Methods). A fixed OFF strain (11168Δ*cj0031*::kan) was generated by constructing a deletion in *cj0031*. Complementation of this mutation (strain 11168-Δ*cj0031-cj0031*c) was achieved by insertion of the wild-type, phase-variable gene into a pseudogene (with expression controlled by a heterologous promoter) and isolation of a variant with an ON number of repeats. Genomic DNA from these strains was digested with a range of methylation sensitive restriction enzymes and tested for differences in the restriction patterns using probes from three different regions of the genome (Figure 3). An obvious difference in the restriction pattern of *cj0031*-ON versus 11168-*cj0031*-OFF and 11168Δ*cj0031*::kan was detected using probe III with inhibition of HindIII digestion in ON but not OFF phase variants or a deletion mutant (Figure 3). Protection of this site was partially restored in the complementation strain, 11168-Δ*cj0031*c, confirming that protection was due to Cj0031, with the incomplete level of protection being due to a sub-optimal expression from the *fdxA* promoter in the complementation vector (36). Additional phase-variants were tested with probe III and exhibited the same restriction pattern (data not shown). Critically, another HindIII site present in Probe I did not show any difference in restriction pattern (Figure 3) suggesting that the putative Cj0031 MTase recognition site did not correspond to but overlapped with one of the two end sequences of the HindIII 5΄-AAGCTT-3΄ recognition sequence in Probe III with inhibition by methylation on the first adenine.

As Cj0031 shares 87% identity with CjeFV of *C. jejuni* strain 81–176 whose recognition sequence was characterized as 5΄-GGGCA or 5΄GGACA (37), we hypothesized that the Cj0031 recognition sequence was GC-rich and had one of three sequences:- 5΄-CCGAA-3΄; 5΄-CGAAG-3΄; or 5΄-CCCGA-3΄ (Figure 2). The alternative was that the site was AT-rich and had a recognition sequence of 5΄AAGAA or 5΄AGAAG. The PSR-HindIII-CCG probe was designed to investigate if the recognition site was 5΄CCGAA or 5΄CGAAG. No inhibition of restriction was observed (Figure 3D). This result indicated that all three cytosine residues were required and that a Cj0031 recognition sites could be 5΄-CCCGA-3΄.

### Detection of the methylation motif of Cj0031 by SMRT sequencing

SMRT is a robust and reliable method for detecting methylation patterns in DNA sequences. The PacBio RS sequencing platform was utilized to generate methylomes for an ON phase variant and deletion mutant *of cj0031* in *C. jejuni* strain NCTC11168 strain. Both genomes contained two degenerate motifs, a palindromic motif (RAATTY) and a non-palindromic motif (GKAAYG; Table 1) that had been previously identified (37) as the recognition sites of CjeNII (Cj1051c), M.CjeNIV (Cj1553c), CjeNIII (Cj0690c) and M.CjeNI (Cj0208). In contrast, a novel methylation motif—5΄CCYGA—was present in the genome sequences for the ON phase variant of Cj0031 but absent from the deletion mutant. This 5΄CCYGA motif, as also observed for 5΄GKAAYG, only exhibited methylation on the adenine of one of the DNA strands. A high level of methylation was observed with 1759 of 1777 sites being detected as having methylation with a mean modification QV of 75%. These results confirmed the modification of 5΄CCCGA in Cj0031 ON phase variants, as detected by Southern blotting, and additionally indicated modification of 5΄CCTGA sites by this enzyme.

**Table 1.** Methylation motifs identified within the genomes of a *C. jejuni* NCTC11168 *cj0031* ON phase-variant and a Δ*cj0031* mutant

Motifs	Modified position	Type	% Motifs detected	# Of motifs detected	# Of motifs in genome	Partner motif
*C. jejuni* NCTC11168 *cj0031* ON						
ACNNNNNCTC	1	m6A	99.91%	1065	1066	GAGNNNNNGT
GAGNNNNNGT	2	m6A	99.25%	1058	1066	ACNNNNNCTC
TAAYNNNNNTGC	3	m6A	99.79%	468	469	GCANNNNNRTTA
GCANNNNNRTTA	3	m6A	97.87%	459	469	TAAYNNNNNTGC
GKAAYG	4	m6A	99.68%	1255	1259	
RAATTY	3	m6A	99.01%	27 011	27 280	RAATTY
CCYGA	5	m6A	98.99%	1759	1777	
*C. jejuni* NCTC11168 Δ*cj0031*						
ACNNNNNCTC	1	m6A	99.91%	1065	1066	GAGNNNNNGT
GAGNNNNNGT	2	m6A	99.25%	1058	1066	ACNNNNNCTC
TAAYNNNNNTGC	3	m6A	99.79%	468	469	GCANNNNNRTTA
GCANNNNNRTTA	3	m6A	97.87%	459	469	TAAYNNNNNTGC
GKAAYG	4	m6A	99.68%	1255	1259	
RAATTY	3	m6A	99.01%	27 011	27 280	RAATTY

### Investigation of the impact of phase variation or mutation of *cj0031* on adherence, invasion and biofilm formation by *C. jejuni*

**Figure 4.**
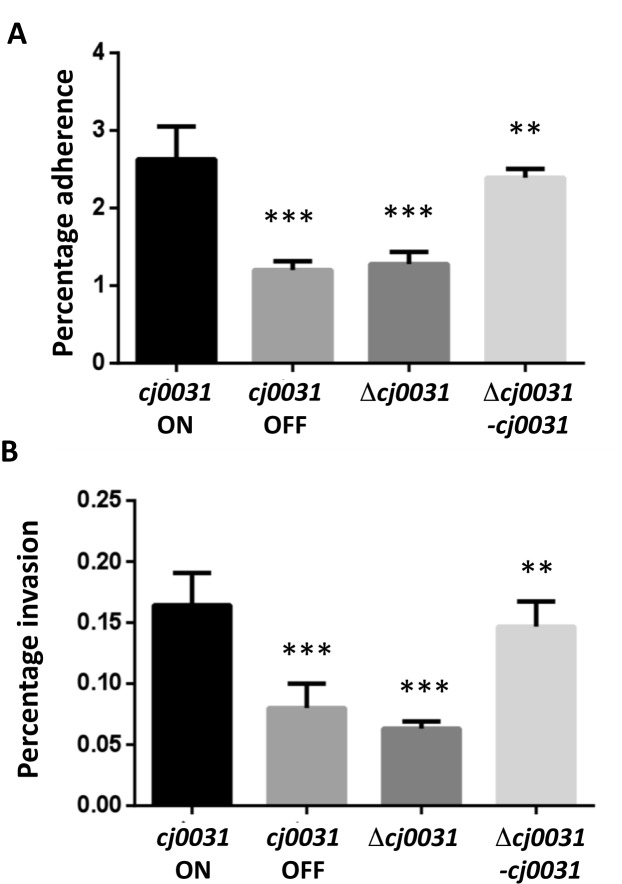
Influence of *cj0031* on adherence and invasion of *Campylobacter jejuni* strain NCTC11168 to Caco-2 cells. Adhesion and invasion experiments were performed using an inoculum of 1 × 10^7^ cells (adherence assay) or 2 × 10^7^ cells (invasion assay) for each bacterial strain and a semi-confluent monolayer of Caco-2 cells. Adherence (panel **A**) was measured after a 4 h incubation as a combination of adherent and invaded bacteria. Invasion (panel **B**) was determined by performing an initial four hour adhesion assay followed by 4 h of gentamycin treatment. In both cases, serial dilutions of total cell lysates were utilized for calculation of the number of cfu/ml and expressed as percentage of the inoculum. Both experiments were performed with an ON and OFF phase-variant of *cj0031* (*cj0031* ON and *cj0031* OFF, respectively), a deletion mutant of *cj0031* (Δ *cj0031*) and a complement of this mutant with *cj0031* inserted into *cj0046*, a pseudogene (Δ *cj0031*–*cj0031*). All of these phase variants or mutants were in a chicken-adapted variant of *C. jejuni* strain NCTC11168. Triplicate readings were utilized for calculation of an SEM (standard error of mean) and error bars. Unpaired Student's *t*-test was used to calculate the *P*-values as compared to *cj0031* ON (**P < 0.01; ***P < 0.001).

Motility is a critical phenotype required for colonization and invasion by *C. jejuni*. As the laboratory strain utilized for the methylome studies is non-motile, phase variants and mutants of *cj0031* were obtained for a hypermotile chicken-adapted variant (i.e. CH11168) of *C. jejuni* strain NCTC11168. The functional consequences of PV or inactivation of *cj0031* on motility were examined using Mueller-Hinton-soft-agar plates. No obvious differences in motilities of these strains were observed, indicating that Cj0031 methyltransferase activity does not affect the motility of *C. jejuni* (Supplementary Figure S1). Having shown these strains to be motile, the adhesion and invasion phenotypes were investigated. In two separate experiments, both an OFF variant and the deletion mutant (i.e. CH11168Δ*cj0031*::kan) exhibited significant 2-fold lower levels of adhesion to Caco-2 cells than an ON variant (Figure 4). Complementation restored the adherent phenotype to the same level as the ON variant. Invasion was assayed using Caco-2 cells and gentamycin-treatment. Both the OFF variant and deletion mutant of *cj0031* exhibited a significant reduction of 2–3-fold as compared to the wild-type *cj0031* ON variant. Complementation of the *cj0031* deletion mutant with an ON variant of the native *cj0031* gene partially restored the invasion phenotype (Figure 4).

**Figure 5.**
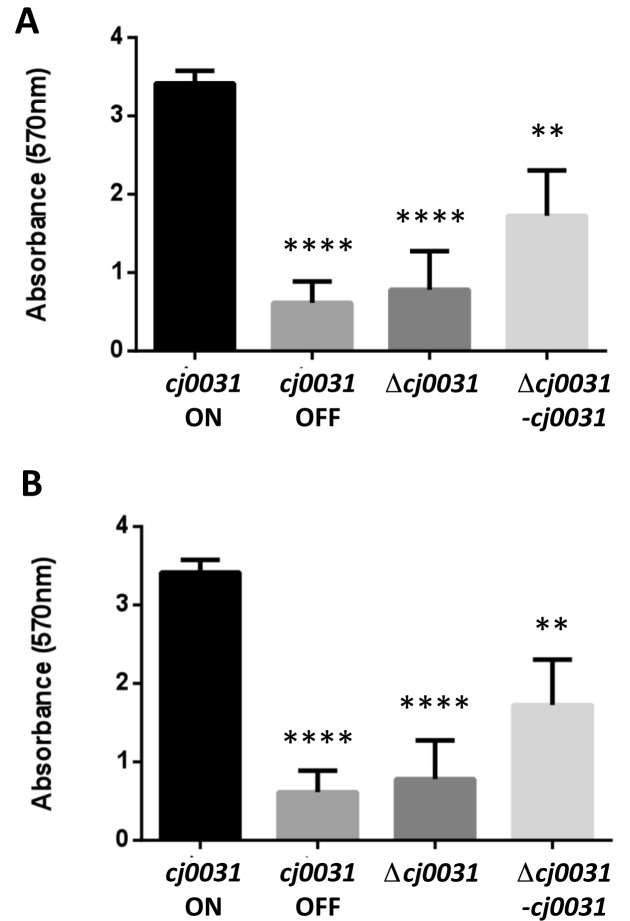
Influence of *cj0031* on biofilm formation by *Campylobacter jejuni* strain NCTC11168. An assessment of biofilm formation was performed using phase variants and mutants of *cj0031* in a chicken-adapted variant of *C. jejuni* strain 11168 (see Figure 4). Biofilm formation was measured by crystal violet (CV) staining of standing cultures in 24-well polystyrene plates following either 48 (panel **A**) or 72 (panel **B**) h incubation at 37°C under micro-aerobic conditions. Quantification of dissolved CV was made at 570 nm. Measurements represent six measurements determined from triplicate readings of two independent experiments. An unpaired Student's *t*-test was used to calculate *P*-values as compared to *cj0031* ON (****P < 0.0001; and **P < 0.01).

Additionally, the contributions of Cj0031 to biofilm formation were examined using a CV staining method (29,40). Both OFF phase-variants and a *cj0031* deletion mutant demonstrated 7- and 4-fold decreases, respectively, in biofilm formation as compared to an ON phase-variant after both 48 and 72 h of growth (Figure 5). The complementation strain (i.e. 11168-Δ*cj0031-cj0031*) partially restored the phenotype to a level that was significantly (*P* < 0.001) different from the deletion mutant, indicating that absence of *cj0031* is deleterious to biofilm formation by *C. jejuni* strain NCTC11168. A further biofilm experiment was performed to test whether PV of *cj0031* or other genes occurred during the course of the biofilm assay. Differences in PV state were assessed for *cj0031* and 26 other loci containing polyG tracts using a multiplex PCR/GeneScan assay (Lango-Scholey *et al.*, in preparation). No differences in the PV state of *cj0031* or any other locus were observed between input and output populations for any of the strains whilst the biofilm phenotype was as observed in Figure 5 (data not shown). One gene, *cj1342*, was in an ON state (i.e. 9G) in the deletion mutant but OFF (i.e. 10G) in the wild-type and complement strains. Phase variants of *cj1342* were isolated for the *cj0031* ON phase variant of the wild-type strain and shown to exhibit identical levels of biofilm formation (data not shown), indicating that the differences in biofilm formation observed in Figure 5 were due to changes in *cj0031* expression and not other phase-variable genes.

**Figure 6.**
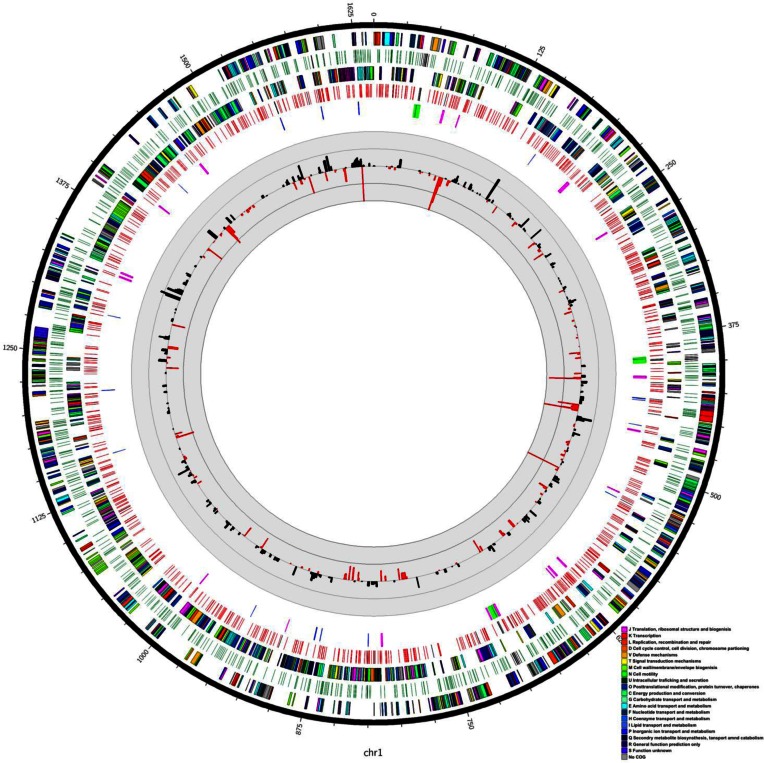
Circos diagram representing the genome of *Campylobacter jejuni* 11168. This diagram was drawn as described by Krzywinski *et al.* (35). The outer ring represents genes transcribed in the forward direction, colour coded according to COG (Clusters of Orthologous Groups function; colour key bottom right). The second ring represents the positions of the CCYGA motif on this forward strand with intergenic sites represented in green and intragenic positions represented in black. The third ring represents genes transcribed on the reverse strand, colour coded according to COG. The fourth ring represents the positions of the CCYGA motif on this reverse strand with intergenic sites represented in red and intragenic positions represented in black. The fifth ring represents the positions of pseudogenes (pink), tRNA genes (blue), rRNA genes (green) on both strands. The histograms show the expression of genes increased in mutant relative to wild-type by more than 1.5-fold (black) or those decreased relative to wild-type by more than 1.5-fold (red). The outer ring of the scale for the histograms represents a 5-10-fold change in expression whilst the inner ring represents a 1.5-5-fold change.

### Transcriptional profiling of Cj0031 phase variants and mutants

Alterations in methylation of Cj0031 recognition sites could impact on transcription of specific genes and produce differences in phenotypic behaviour. RNASeq profiles were generated for an ON variant in strain NCTC11168 and the deletion mutant of *cj0031*. Examination of sequences generated from the RNA-Seq data confirmed that *cj0031* contained an ON number of repeats and that the mutant lacked the gene. Major differences in gene expression were observed with 89 and 130 genes exhibiting a statistically-significant reduction or increase, respectively, of >1.5-fold in the mutant (Figure 6). A subset of 20 genes exhibited changes of >5-fold (Figure 6; with 14 having reduced and 6 increased expression). An qRT-PCR of nine genes confirmed the observed changes in relative gene expression levels (Supplementary Figure S2). Major changes in gene expression were observed in two tRNA genes and genes whose products contribute to metabolism (e.g. Uxa, altronate hydrolase; Cj0073-Cj0075, iron-sulphur cluster proteins) or transport (e.g. PutP, a sodium-proline symporter; HydA*2*, NI-Fe hyrdrogenase; Lct*P*, L-lactate permease). Only one of nine genes (i.e. *capA, cadF, pldA, peb1A, flpA, ciaB, cipA, cj0268c* and *cj1349*) with evidence of roles in adhesion and invasion, was altered in expression with a 3-fold increase in expression of *peb1A*.

Altered expression of genes in Δ*cj0031* may be due to differential methylation of 5΄CCYGA sequences. The NCTC11168 genome contains 1776 of these sequences with an average of 1.1 sites per kbp and ∼1 site per gene. Only 69 sites are located in intergenic regions and these sites are associated with 38 genes. Significant >1.5-fold alterations in expression were only observed for three of these genes in Δ*cj0031* (two upregulated and one downregulated). None of these genes had known roles in adhesion, invasion or biofilm formation. The loci that contained genes exhibiting large differences (>5-fold) in expression were examined for the presence of Cj0031 methylation sites. In some of these loci, 2–4 5΄CCYGA sequences were present within the reading frames of multiple genes but in other loci most of the genes contained 0–1 sequences (Supplementary Figure S3). As an alternative, loci with 4–6 genic 5΄CCYGA sequences were examined for alterations in gene expression and multiple examples were observed with <1.5-fold changes in expression. There was however an association between the presence of multiple recognition sites and an increase in gene expression with 23% of genes containing four or more repeats (*n* = 83) exhibiting a significant increase in expression whereas only 7% and 10% of genes with 1–3 (*n* = 1038) or 0 (706) sites, respectively, exhibited an increase in expression. However the average increase in expression for genes containing four or more sites was 2-fold with no genes showing a >3.5-fold increase in expression. Overall these results indicated that there is only a weak association between alterations in gene expression in Δ*cj0031* and the presence of 5΄CCYGA sequences.

### Assessment of the phage restriction activity of Cj0031

The classic activity of RM enzymes is to prevent phage replication. Stocks of *Campylobacter* bacteriophages propagated on *C. jejuni* strain PT14 were tested for plaque formation at the routine test dilution (log_10_ 6 PFU) on bacterial lawns of *cj0031* phase-variants and the deletion mutant. Some of these bacteriophages exhibited plaque formation on the *cj0031* OFF variant or deletion mutant but not on *cj0031* ON. These phages were selected for further study in order to determine whether they were subject to restriction by the putative endonuclease activity of Cj0031. Phages were propagated on Δ*cj0031* to allow for methylation on sites for all of the NCTC11168 RM systems except *cj0031* and then tested for growth on *cj0031* phase-variants and mutants. The efficiency of plating (EOP) values of the deletion mutant and *cj0031* OFF variant were similar, whereas the EOPs recorded for the *cj0031* ON variant exhibited between 5- and 1000-fold reductions in PFU (Table 2). These data are consistent with the hypothesis that *cj0031* encodes a functional endonuclease.

**Table 2.** Relative efficiency of plating of phages on phase variants and mutants of cj0031 in *C. jejuni* strain NCTC11168

Phage^a^	Relative EOP^b^		
	*cj0031* ON	*cj0031* OFF	Δ*cj0031*
CP8	0.05	1	1
CP25	0.04	1	1
CP30	0.01	1	1
A1b	0.2	1	1
X3	0.1	1	1
G3	0.01	0.8	1
G4	0.06	0.9	1
MC1a	0.008	0.8	1
11	0.009	0.9	1
19b	0.005	0.9	1
NCTC12671	0.001	1	1
NCTC12679	0.02	0.8	1

^a^Phages were propagated on 11168Δcj0031;

^b^EOP of the sensitive phages on the test host relative to a non-restricting host.

The potential of an RM system to restrict phage replication is dependent on the presence and frequency of recognition sites in phage genomes. The methylation site frequencies were determined for all of the *C. jejuni* RM systems of ten phages whose genome sequences were available and ranged in size from 132 to 178 kb (Table 3). The Cj0031 site was present in all phage genomes at similar frequencies including phages with sensitivity (i.e. CP8 and CP30) to restriction on Cj0031 ON variants. A comparison between enzymes indicated that the highest site prevalence was for the AT-rich recognition site of Cj0208 (3–4 -sites/kb for RAATTY) whilst both Cj0031 and Cj0690 exhibited frequencies of ∼1/kb whereas the more complex sites of Cj1051 and Cj1553 were present at <0.5 sites/kb.

**Table 3.** Prevalence of recognition sites for the RM systems of *C. jejuni* strain NCTC11168 in phage genomes

Phage^a^/Group^b^	Cj0208		Cj1553c		Cj0031		Cj1051c		Cj0690c	
	Tot^c^	Fre^d^	Tot	Fre	Tot	Fre	Tot	Fre	Tot	Fre
CP220/II	665	3.7	59	0.3	175	1.0	75	0.4	163	0.9
CPt10/II	668	3.8	59	0.3	177	1.0	73	0.4	160	0.9
CP21/II	713	3.9	66	0.4	183	1.0	75	0.4	167	0.9
CP81/III	403	3.0	57	0.4	149	1.1	79	0.6	152	1.1
NCTC12673/III	394	2.9	56	0.4	152	1.1	80	0.6	165	1.2
CPX/III	408	3.1	57	0.4	151	1.1	76	0.6	147	1.1
CP30A/III	388	2.9	53	0.4	142	1.1	75	0.6	154	1.2
CP8/III	408	3.1	57	0.4	151	1.1	76	0.6	147	1.1

^a^Accession numbers: FN667788, FN667789, HE815464, FR823450, GU296433, JN132397, JX569801, JK148616;

^b^Group, phages belonging to group II have genomes of 180–190 kb whilst those in group III have genome sizes of 130–140 kb (52,53);

^c^Tot, total number of sites per genome;

^d^Fre, frequency of sites per kb.

## DISCUSSION

*Campylobacter jejuni* contains several phase variable genes with known or potential roles in survival and adaptation of this species to various niches in hosts and the environment. While phase-variable genes usually encode surface structures, a provocative association with non-surface coding genes is widely reported and in particular a distinctive PV signal is found for RM systems of several pathogenic bacteria (19–22,41). PV of surface molecules is an adaptive strategy enabling escape of not only innate and adaptive host immune responses but also a dynamic and evolving phage population (8,9). The phase-variable RM systems provide another strategy to escape selection by phages. A more surprising finding was of a phasevarion whereby PV of the methyltransferase of a phase-variable RM system coordinates the expression of multiple genes through changes in genome methylation (19). We define herein the recognition sites of a novel, phase-variable Type IIG RM system and investigated the contributions of this enzyme to a *Campylobacter* phasevarion and phage restriction.

Previous SMRT sequencing of *C. jejuni* strain NCTC11168 detected methylation sites for four of the putative RM systems but not for Cj0031 as this gene was in an OFF state in the analysed variant of this strain (37). We isolated an ON variant and constructed both a deletion mutant and complementation strain to facilitate characterization of this RM system. By examining inhibition of methylation activity, we detected a HindIII site that exhibited a differential pattern of digestion between ON and OFF variants of *cj0031*. This suggested that the Cj0031 recognition site was either 5΄CCCGA with methylation on the adenine or an AT-rich sequence (e.g. 5΄AAGAA). SMRT sequencing identified the methylation site of this enzyme as either 5΄CCCGA or 5΄CCTGA as methylation of these sites was absent in a *cj0031* deletion mutant but present in an ON phase variant of this gene.

The phylogenetic analysis of *cj0031* showed that homologues of *cj0031* were present in *H. pylori*, a close relative to *C. jejuni*, as well as several other more diverse bacterial species. Homologues of *cj0031* were present in other strains of *C. jejuni* and have a 90–99% sequence similarity with conservation of the motifs characteristic of restriction endonucleases. Despite this high overall level of conservation, one region (IV; Figure 1) of the protein exhibited high levels of variation between all alleles of Cj0031 for which methylomes have been determined. This region is predicted to encode the TRD as has been predicted for the hypervariable regions of other RM-associated methyltransferases. The methylation site of CfeFV, the homologue of *cj0031* in strain 81–176, has been characterized by SMRT sequencing as 5΄GGRCA (37) whereas that of Cj0031 was 5΄CCYGA. The putative TRD regions of these homologues differ by 87–91 amino acids in the 136–140 residues of region IV suggesting that the amino acids responsible for site-specific DNA recognition are located therein. The IA3902 homologue, CjeIAORF32P, exhibits significant amino acid variation from Cj0031 in both regions III and IV (Figure 1) and methylates at a 5΄GAAGAA sequence (38). A notable feature of these alignments is of the absence of the polyG tract in the other Cj0031 homologues suggesting that PV may be restricted to a sub-set of alleles.

Populations of *C. jejuni* strain NCTC11168 will contain phase-variants of Cj0031 with either methylated (ON) or non-methylated (OFF) 5΄CCYGA sequences, raising the potential for association of a phasevarion with PV of Cj0031. A phasevarion is a set of genes whose expression is regulated by ON-OFF switching of site-specific methylation by a phase-variable methyltransferase. The observation of reductions in adhesion, invasion and biofilm formation in *cj0031* deletion mutants was indicative of association of Cj0031 PV with a phasevarion. These alterations in host cell attachment were not due to differences in motility (Supplementary Figure S1) but were in general minor differences in phenotype. The degree of these effects could have been reduced by PV of the ON variants so that starting populations contained variable proportions of OFF variants. An alternative explanation of differences in phenotype is the impact of PV of other genes. The potential importance of this caveat was reduced by the observation of similar phenotypes for the ON variant of the wild-type and complementation mutant and similarly for the OFF variant and deletion mutant. Furthermore, no influence on biofilm formation of PV of *cj0031* during the experiment or of differences in expression of other phase-variable genes between strains was observed in additional repetitions of the biofilm assay. Inactivation of another putative methyltransferase, Cj1461, reduced both the motility and invasion by *C. jejuni* strain 81–176 (42). Similarly, Fields and Thompson (43) reported that inactivation of CsrA, a global post-transcriptional regulator, reduced motility, biofilim formation and adherence of *C. jejuni* strain 81–176 but enhanced invasion leading to the proposal that CsrA directly or indirectly represses invasion specific genes. The RNASeq analysis of Δ*cj0031* detected an altered level of expression for Peb1A (*cj0921c*, −2.8-fold) but not for any other known adhesins or invasins. Peb1A is a periplasmic protein that mediates uptake of dicarboxylic amino acids (44). A variable association of this gene with reductions in adhesion has been reported (45). Thus, the Cj0031 methyltransferase might act as a phasevarion with alterations in expression of Peb1A resulting in reductions in host cell adhesion and invasion. Alternatively the alterations in metabolic pathways, also observed in the Δ*cj0031* RNASeq data, could be responsible for the observed phenotypic variation. A final possibility is that observed differences in gene expression may have impacted on other types of phenotypic variation.

The proposed mechanism of regulation of a phasevarion is through differential methylation of promoter elements leading to alterations in binding of regulatory proteins and/or RNA polymerase to promoter elements in an analogous manner to Dam methylase-mediated regulation of bacterial gene expression (17 and 18). There was only weak evidence of a genome-wide association between presence of 5΄CCYGA sequences and alterations in transcription as detected in the RNASeq data. An alternate possibility is that variable methylation at only some of the 5΄CCYGA sites has any effect on transcription and that the differential regulation is through perturbation of one or more global regulators of gene expression. However, there were no significant alterations in expression of known two-component regulators (i.e. RacRS, DccRS, cbrRS and FlgRS), sigma factors (FliA and RpoN), CsrA or of Fur (a significant 2-fold change in *flgR* was observed but this was disregarded as there was no overall change in expression of the *fla* genes). Finally, the Peb1A gene contained a single 5΄CCYGA sequence within the reading frame, which is the number expected by chance for any gene of this genome. Thus, neither the overall nor specific distribution of Cj0031 methylation sites provides a clear insight into the mechanism for altering gene expression as implied by differences in phenotypic variation or as detected in the RNASeq data.

An alternate putative function of all RM systems is restriction of phage replication. Testing of a phage panel revealed that inactivation of *cj0031* resulted in a higher EOP for 12 phages. This loss of inhibition of phage replication provides evidence that Cj0031 is an active restriction endonuclease with the ability to restrict phage DNA. The efficiency of restriction by RM systems is, for some systems, directly proportional to the number of recognition sites located in the genome of the invading element (46–48) with low numbers of restriction sites for a specific RM system correlating with a greater probability to escape restriction. The high prevalence of sites for Cj0031 in two of the restricted phages, CP8 and CP30, supports the view that the restriction activity is correlated with the number of restriction sites. Intriguingly, the *cj0031* site has a higher prevalence (1.5- to 6-fold) in all phage genomes than that of the homologue present in strain PT14 (i.e. A911_00150; GAACAA). If the main function of Cj0031 is restriction of phage growth and the number of recognition sites is correlated with higher restriction of phage growth, then the Cj0031 and CfeFV alleles may be more widely distributed in *Campylobacter* genomes than the alleles with 6 bp recognition sites. Evolution of PV of Cj0031 could then be driven by selection for phage resistance in the ON state and selection for expression of one state of the phasevarion in the alternate state. Alternatively selection for the OFF state may be driven by receptiveness to adaptive mutations associated with natural transformation (assuming in-coming DNA can be restricted by Cj0031 endonuclease activity) or fluctuating levels of Cj0031-resistant methylated phage genomes as speculated elsewhere (49).

Our study provides evidence of a dual role for PV of the Type IIG enzyme, Cj0031, of *C. jejuni* strain NCTC11168 as a limiter of phage spread and a regulator of a gene expression. The underlying mechanism of action for the alteration in gene expression is not known and does not appear to involve differential methylation of specific recognition sites by Cj0031. Nevertheless PV of Cj0031, as detected during infections in chickens and mice (8,50), produces changes in expression of multiple genes that may lead to phenotypic variation and generation of highly differentiated variants with differing capabilities of adaptation to diverse niches.

## Supplementary Material

Supplementary Data

SUPPLEMENTARY DATA
