# Supplementary material for: Phase variation of a Type IIG restriction-modification enzyme alters site-specific methylation patterns and gene expression in Campylobacter jejuni strain NCTC11168
Source: Nucleic Acids Res. 2016 Jan 18;44(10):4581–94. doi: 10.1093/nar/gkw019 (PMC4889913; doi:10.1093/nar/gkw019)
Supplement: Supplementary Data [file gkw019_Supp.doc]

### Supplementary Table SI. Oligonucleotide sequences of primers used for cloning and generation of probes

| **Name** | **Sequence (5’-3’)** | **Purpose** |
| --- | --- | --- |
| 3544-for | TGTGCGTGAAGGTTTGATCG | Probe I |
| 4029-rev | gatccatcaacatccgcatc |
| 425130-for | TTCCGTGCCTTGGCTCCTG | Probe II |
| 425852-rev | TACAGCAGGTATGGCAAGTG |
| 1036450-for | TTTAATCTCGTATCTTAGAC | Probe III |
| 1036809-rev | AAGCAAAACACATTCTTCATC |
| PSR1-Hind-F | GTGCAAAGCAATAAACCTGAACTTTTAA | PSR-HindIII-CCG |
| PSR1-Hind-R | CTGCTAAAGAACAAGCTTCATAAGAAAT |
| 0031-2108-F-pst1 | AACTGCAGAATTCTCATCACGGGTAG | Deletion mutant construction |
| 0031-2832-R-2832-Bam | CGGGATCCATCATCGTCCAAATTTC |
| 0032-Bam-F | CGGGATCCAAGATAGAACGCATAGG |
| 0032-R | CGGGATCCGCGGTACCGTGTATTTGTTAGAAGTG |
| N-cj0031-F | AAACGTCTCACATGCATTTCACTTTGCTAATGAAAAAG | Construction of complementation mutant |
| N-cj0031-R | TTTCCATGGTTCTTCCATTATTTCCCTTCTATG |
| CAT-INV-F | GCGGTCCTGAACTCTTCATGTC | Checking of mutants/complement |
| 0031-2108-R | CATCTTTTAGCACGGTGTTTTA |
| 0046-F | CTCTCTCCFCTAGAAATTAAATCC |

### Supplementary Table SII. Source of bacteriophages exhibiting differential plaque formation

| **Bacteriophage** | **Source/Accession** |
| --- | --- |
| CP8 | UN^a^/ KF148616 |
| CP25 | UN |
| CP30A | UN/ JX569801 |
| A1b | UN |
| X3 | UN |
| G3 | UN |
| G4 | UN |
| MC1a | UN |
| 11 | UN |
| 19b | UN |
| NCTC12671 | NCTC^b^ |
| NCTC12679 | NCTC |

UN^a^, university of Nottingham stocks; NCTC^b^ , National Collection of Type Cultures

**Supplementary Table SIII**. Accession numbers for PacBio data

| Sample | Study accession | Sample accession | Experiment accession | Run accession | Submission accession |
| --- | --- | --- | --- | --- | --- |
| NCTC11168 Cj0031 ON | ERP008529 | ERS572885 | ERX808962 | ERR791723 | ERA420037 |

| NCTC11168 Cj0031 ON | ERP008529 | ERS572885 | ERX930417 | ERR849506 | ERA428794 |
| --- | --- | --- | --- | --- | --- |

| NCTC11168 Cj0031 KO | ERP008529 | ERS572886 | ERX808963 | ERR791724 | ERA420037 |
| --- | --- | --- | --- | --- | --- |
| NCTC11168 Cj0031 KO | ERP008529 | ERS572886 | ERX930418 | ERR849507 | ERA428794 |

## Supplementary Table SIV. Primer pairs utilised in qRT-PCR to determine the change in expression for selected genes.

| **Locus Tag**  **A911_** | **Target Gene** | **Forward Primer** | **Reverse Primer** | **Annealing Temperature (°C)** |
| --- | --- | --- | --- | --- |
| Cj1186 | *pet*A | AGCTCTCCATCTTGCATTCC | CAAGTGTCAAAGCTGCAGG | 57 |
| Cj0169 | *sod*B | AAACTTCAAATGGGGGCGTAT | CACAGCCACAGCCTGTAC | 57 |
| Cj1491 | *che*Y | AATCTCTCTTGCCATATCCAG | AATCGGAGCTCAAAATGGAG | 57 |
| Cj0074 | *-* | GCCCAATGCACCTTAATGC | GACTGGCAGGGCTTAAGAG | 57 |
| Cj0509 | *clp*B | GCACCTAAGAGTCTTGAAACA | ACAATCGGCTAAAGCTTTGG | 57 |
| Cj0145 | - | CTGTTAGTGCAAGCACACAAG | CTAAGATACTCACACCCACACTG | 57 |
| Cj1220 | *gro*ES | AAACAACAGCCTCAGGCATAA | TTCTGTTCCACCGTATTTAGCA | 57 |
| Cj1402 | *pgk* | AAGTCTAGCAAGACGCTTAGC | TAGACGCATAAGATCAGCTATTCC | 57 |
| Cj1601 | *his*A | ACCATGCAAGGAGTAAATGTAA | CCTACGATAACCCCACTACAA | 57 |
| Cj0547 | *fla*G | ATTCAAACCGATAGAAGTCAAG | ATATCAGCCAACTTTTCACTCA | 57 |


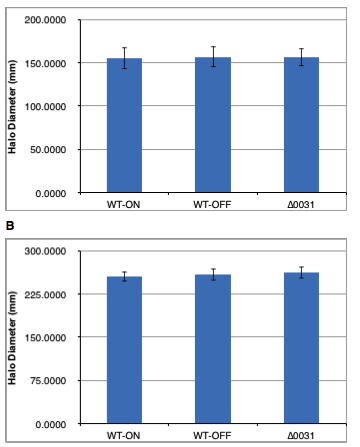


Supplementary Figure S1. Effect of *cj0031* mutation on motility of *C. jejuni*. Motility was determined by inoculating strains on to motility plates and visually determining movement after 48 hours incubation at 37^o^C by measuring spread from the initial inoculation point (halo diameter). Data are the means calculated from duplicate readings from three experiments. Bars represent standard deviation. WT-ON, CH11168-cj0031-ON; WT-OFF, CH11168-cj0031-OFF; D0031, CH11168Dcj0031kan.


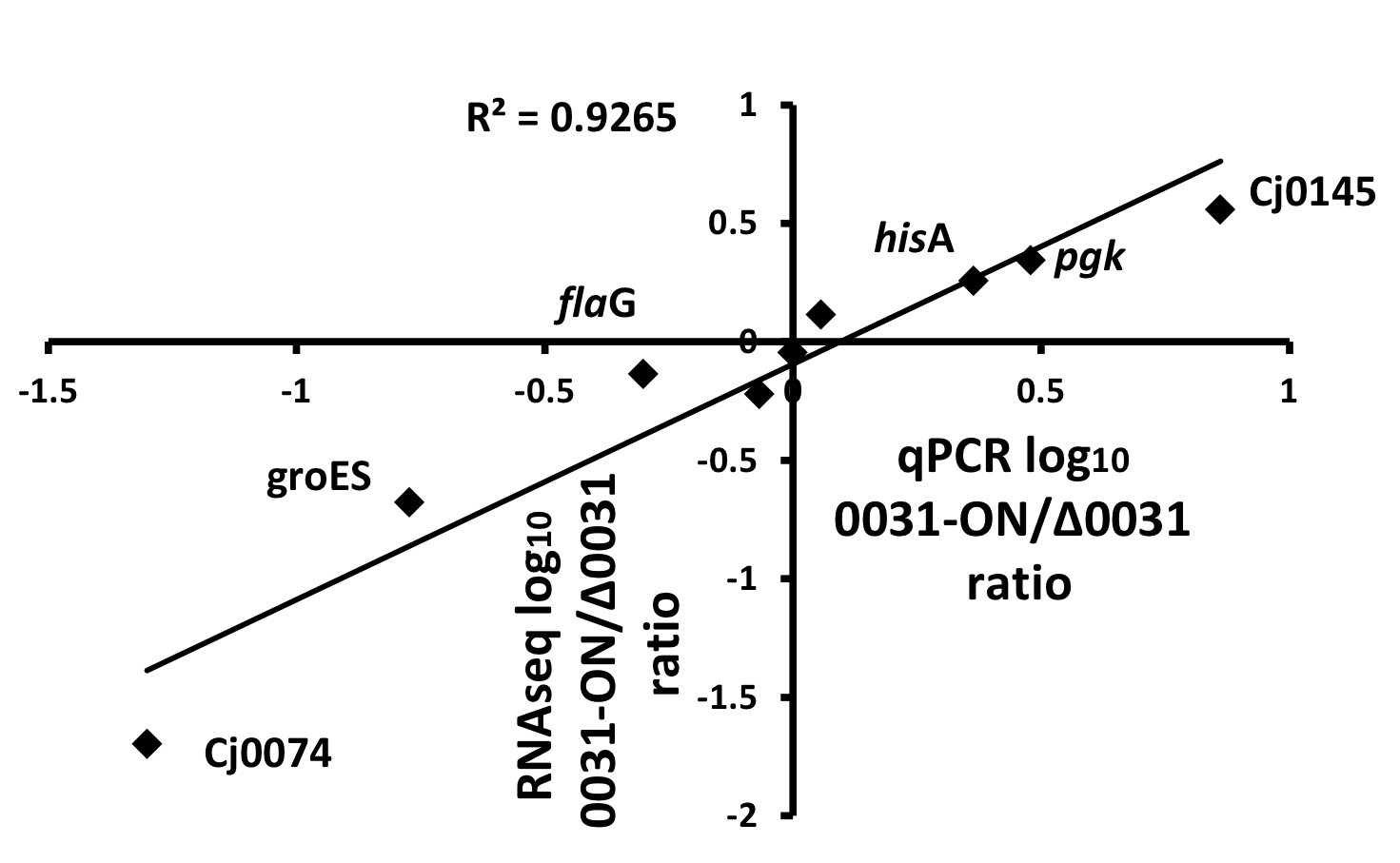


Supplementary Figure S2. RT-qPCR comparison of relative expression for specific genes in a C. jejuni strain 11168 phase variant and mutant of *cj0031*. 0031-ON, NCTC11168 cj0031ON phase-variant; Δ0031, 11168-Δcj0031::kan.


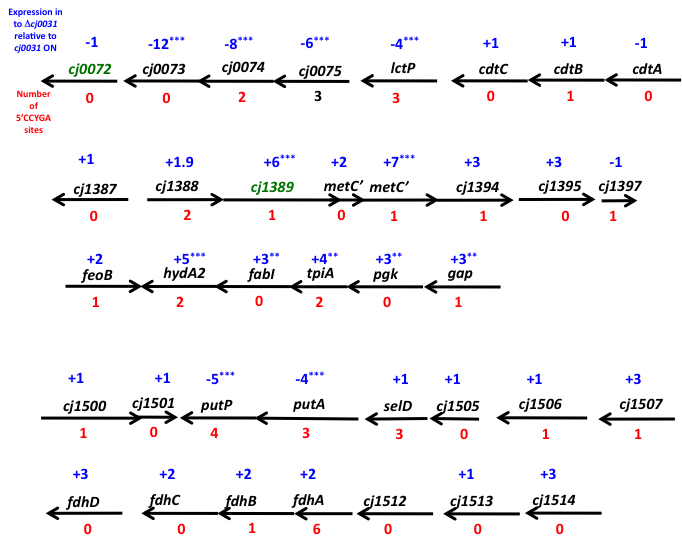


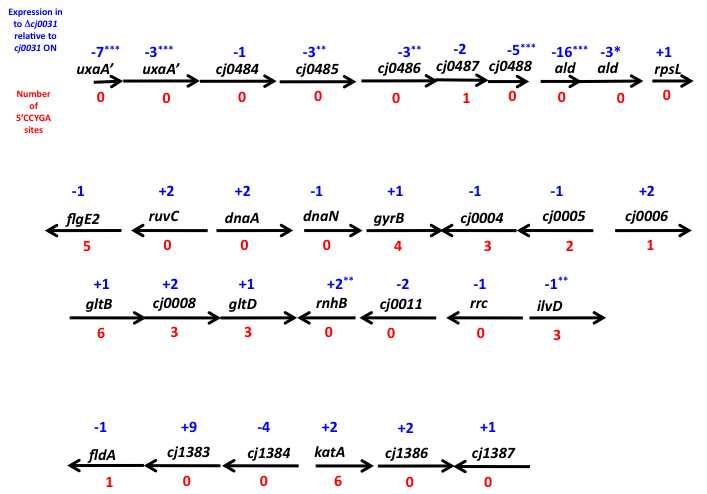


Supplementary Figure S3. Gene-by-gene comparisons of the differences in gene expression due to inactivation of *cj0031* with the presence of methylation sites for Cj0031. The order and orientation of genes is shown for several loci in the *C. jejuni* NCTC11168 genome with each gene being represented by an arrow. The figure above the gene is the fold-difference in expression level for 11168Δcj0031 as compared to an ON phase-variant of the wild-type 11168 strain as detected using RNA-Seq. The asterisk indicates ranges for the normalized, FDR-corrected p value as determined using the Baggerley’s test; *, 0.05-0.01; **, 0.01-0.001; ***, >0.001. Note that values without an asterisk were not significant. The figure below the line is the combined number of 5’CCCGA and 5’CCTGA sites present within the coding region of each gene. Genes highlighted in green are pseudogenes.
