# Supplementary material for: Phase variation of a Type IIG restriction-modification enzyme alters site-specific methylation patterns and gene expression in Campylobacter jejuni strain NCTC11168
Source: Nucleic Acids Res. 2016 Jan 18;44(10):4581–94. doi: 10.1093/nar/gkw019 (PMC4889913; doi:10.1093/nar/gkw019)
Supplement: SUPPLEMENTARY DATA [file supp_44_10_4581__index.html]

Phase variation of a Type IIG restriction-modification enzyme alters site-specific methylation patterns and gene expression in Campylobacter jejuni strain NCTC11168 — Phase variation of a Type IIG restriction-modification enzyme alters site-specific methylation patterns and gene expression in Campylobacter jejuni strain NCTC11168 — SUPPLEMENTARY DATA 

# Phase variation of a Type IIG restriction-modification enzyme alters site-specific methylation patterns and gene expression in *Campylobacter jejuni* strain NCTC11168

## SUPPLEMENTARY DATA

- SUPPLEMENTARY DATA
